# COVID-19 Pandemic Care Service Changes and Advance Care Planning on Death Anxiety Among Family Caregivers

**DOI:** 10.1093/geroni/igaf122.2606

**Published:** 2025-12-31

**Authors:** Jung-Nim Kim

**Affiliations:** Tokyo University of Social Welfare, Isesaski City, Gumma, Japan

## Abstract

**Background:**

The number of family caregivers encountered significant difficulties accessing healthcare services and support from relatives during the COVID-19 pandemic due to changes in care service availability at home. Although appropriate support may reduce death anxiety and improve caregiving capacity at home, few studies have examined this relationship. This study investigated the direct associations between COVID-19 related care service changes and Advance care planning (ACP) on family caregivers’ death anxiety, as well as indirect associations through decreased in care services during COVID-19 and ACP implementation.

**Methods:**

Interview surveys were conducted in 2021 and 2023 in areas near the Tokyo Metropolitan region with family caregivers of community-dwelling older adults who were certified as requiring care under Japan’s long-term care insurance system. This study analyzed the combined data from each survey (n = 1,057).

**Results:**

Approximately 18.9% of family caregivers reported decreased care services during COVID-19. SEM analyses revealed that changes in care services due to COVID-19 and ACP directly influenced death anxiety. Care recipients’ ADL and economic situation revealed indirect effects through ACP, but did not show any indirect effects through changes in care services during COVID-19. Multigroup SEM analyses indicated that family caregivers who engaged in ACP communication experienced more decreased in care services during COVID-19.

**Conclusions:**

Family caregivers who engaged in ACP communication and experienced reduced care services during COVID-19 may be at higher risk of death anxiety without adequate support at home. Developing strategies and policies to make home-based healthcare services and support is essential to mitigate this risk.

